# Clinical Effectiveness of Clear Aligner Therapy During Interceptive Orthodontic Treatment in Adolescent Patients: A Systematic Review and Meta-Analysis

**DOI:** 10.7759/cureus.88157

**Published:** 2025-07-17

**Authors:** Diya Chakraborty, Nivedita Sahoo, Bhagabati Dash, Biswaroop Mohanty, Sanghamitra Jena

**Affiliations:** 1 Orthodontics and Dentofacial Orthopaedics, Kalinga Institute of Dental Sciences, Kalinga Institute of Industrial Technology (Deemed to be University), Bhubaneswar, IND

**Keywords:** adolescent orthodontics, arch expansion, clear aligner therapy, interceptive orthodontics, invisalign first, mixed dentition

## Abstract

Interceptive orthodontics plays a crucial role in guiding dental and facial development while addressing occlusal discrepancies at an early stage. Clear aligner therapy (CAT) has gained popularity as an alternative to conventional orthodontic techniques, particularly with the introduction of Invisalign First for treating patients in the mixed dentition phase. This systematic review, conducted in accordance with the Preferred Reporting Items for Systematic Reviews and Meta-Analyses (PRISMA) guidelines, assesses the effectiveness of CAT in adolescent interceptive orthodontics. A thorough literature search across PubMed, Embase, and Scopus led to the identification of eight relevant studies examining treatment outcomes such as arch expansion, anterior crossbite correction, and patient satisfaction. The search strategy employed keywords and MeSH terms such as “clear aligners”, “interceptive orthodontics”, “adolescents”, “mixed dentition”, “arch expansion”, and “anterior crossbite”, combined using Boolean operators like AND and OR to ensure comprehensive coverage.

The findings suggest that CAT facilitates transverse arch expansion, delivers predictable clinical results, and promotes better patient adherence. Satisfaction levels among both patients and parents ranged between 85% and 92%, with clear aligners being preferred over traditional orthodontic appliances due to their enhanced comfort and aesthetic appeal. However, limitations such as small sample sizes and inconsistencies in study methodologies restrict the generalizability of these results. A meta-analysis indicated moderate success in arch expansion (3.0-3.5 mm), though significant heterogeneity was observed (I² ~99.9%). While clear aligners show considerable promise in early orthodontic intervention, further well-structured, large-scale studies are necessary to validate their effectiveness and optimize treatment protocols.

## Introduction and background

Interceptive orthodontics is a proactive approach aimed at optimizing dentofacial development and addressing occlusal issues during the transitional phase from primary to permanent dentition. By intervening early, it can simplify or even reduce the need for more extensive orthodontic treatment in the future, making it a crucial component of preventive dental care. It involves early identification and correction of problems such as spacing, crowding, bite irregularities, and abnormal tooth eruptions [1].

While traditional fixed appliances have been the mainstay, clear aligners are increasingly favored in mixed dentition due to advancements in material science and digital technology [2]. Since its introduction in 1997, Invisalign (Align Technology, Tempe, AZ) has significantly transformed orthodontic care, initially targeting mild to moderate malocclusions in adults [3]. In 2008, the launch of "Invisalign Teen" extended its application to younger patients by incorporating eruption wells to accommodate emerging permanent teeth [4]. However, its use remained limited to minor cases.

A major leap occurred in 2019 with the introduction of "Invisalign First," specifically designed for early interceptive treatment. While Invisalign has been a pioneer in this field, other aligner systems such as Angel Aligner, Smartee, and Spark Aligners have also developed solutions tailored for interceptive orthodontics, contributing to the expanding scope of clear aligner therapy in growing patients. This system featured improved eruption wells, tools for arch expansion, and enhanced software algorithms for better control of developing dentition [3]. These innovations enabled the management of more complex issues, including crossbites and guided tooth eruption.

Clear aligners offer several advantages over traditional braces, including improved periodontal health, reduced discomfort, easier hygiene maintenance, and minimal impact on daily activities [4]. Clinically, they provide efficient maxillary arch expansion and effective management of transverse discrepancies, with more controlled and predictable outcomes and less molar tipping than conventional expanders.

Class II corrections with functional appliances often face limitations due to compliance and mechanical imprecision. New solutions, such as the Runner system and Align Technology’s mandibular advancement features, integrate jaw repositioning with aligner therapy [5]. These advancements support progressive mandibular shifts, enhance skeletal development, and streamline treatment protocols. For example, systems like Invisalign with mandibular advancement (MA) utilize precision wings built into the aligners to posture the mandible forward incrementally, mimicking the effects of functional appliances. This dual mechanism allows simultaneous correction of class II skeletal discrepancies and dental alignment, reducing the need for separate phases of treatment and improving patient compliance in growing individuals.

Patient-reported outcomes indicate higher satisfaction among adolescents and parents using clear aligners, highlighting greater comfort, less pain, and a positive impact on quality of life. With growing clinical evidence and ongoing innovation, clear aligner therapy is poised to become a cornerstone of modern interceptive orthodontics. Its expanding capabilities highlight a significant shift in the orthodontic landscape, toward less invasive, more patient-centric treatments that prioritize comfort, aesthetics, and early intervention. This evolution has the potential to not only improve clinical outcomes but also enhance patient cooperation and long-term oral health.

## Review

Materials and methods

This systematic review followed the Preferred Reporting Items for Systematic Reviews and Meta-Analyses (PRISMA) guidelines to evaluate the effectiveness of clear aligner therapy (CAT) in adolescent interceptive orthodontic treatment. The research question was: "How effective is clear aligner therapy during interceptive orthodontic treatment in adolescent patients?" This review specifically aimed to evaluate both clinical effectiveness, including outcomes such as arch expansion, crossbite correction, mandibular advancement, etc., and patient-centered outcomes like satisfaction and comfort.

The null hypothesis stated that CAT has no clinical effectiveness in adolescents, whereas the alternate hypothesis stated that CAT impacts interceptive orthodontic treatment outcomes in adolescents. The review was registered with the International Prospective Register of Systematic Reviews (PROSPERO), ensuring adherence to a structured methodology. The literature search was guided by the PICO (participants, intervention, comparison, outcome) framework, which defined the population as adolescents (10-19 years) undergoing interceptive orthodontic treatment. The intervention involved the use of CAT, while the comparison included conventional myofunctional appliances or fixed/removable orthodontic devices. The outcomes assessed encompassed both clinical parameters, such as arch expansion, anterior crossbite correction, and mandibular advancement, as well as subjective measures, including patient satisfaction, appliance acceptability, and practitioner perspectives. This comprehensive approach allowed for an inclusive evaluation of CAT’s effectiveness in adolescent interceptive orthodontics.

A systematic search was conducted across PubMed, Embase, and Scopus databases for studies published between January 2019 and February 2024. The search strategy incorporated a combination of MeSH terms and keywords, including “clear aligners”, “interceptive orthodontics”, “adolescents”, “Invisalign First”, “arch expansion”, and “anterior crossbite”, using Boolean operators (AND/OR) to maximize search sensitivity. The initial search yielded a total of 19 records. After removing two duplicates, 17 unique articles were screened based on titles and abstracts. Studies were included if they were published between January 2019 and February 2024, written in English, and involved adolescent patients aged 10-19 years undergoing interceptive orthodontic treatment using CAT. Eligible studies reported either clinical outcomes, such as arch expansion, crossbite correction, incisor alignment, or mandibular advancement, or patient- and practitioner-reported outcomes like satisfaction, acceptability, and compliance. Both interventional and observational study designs were considered, including randomized controlled trials, cohort studies, cross-sectional studies, case series, and retrospective analyses. Exclusion criteria included studies involving adult populations, treatments unrelated to the interceptive phase, non-English publications, and articles not available in full text. Additionally, review articles, conference abstracts, editorials, and letters to the editor were excluded, as were studies that did not report clear aligner use as a primary intervention or lacked outcome data relevant to interceptive orthodontic objectives.

Search filters were applied to limit results to English-language, full-text, human studies. No restrictions on study design were applied during the initial screening. To ensure relevance, only studies addressing malocclusions typically treated in the interceptive phase, such as posterior crossbites, anterior crossbites, class II malocclusions, and transverse deficiencies, were included.

**Figure 1:**
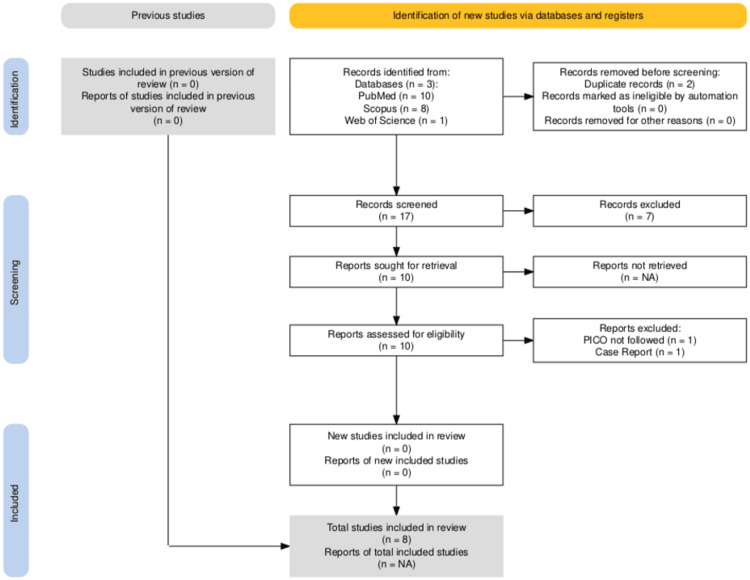
PRISMA flowchart representing the steps followed for the search and screening. PRISMA: Preferred Reporting Items for Systematic Reviews and Meta-Analyses; PICO: participants, intervention, comparison, outcome.

Two independent reviewers (Author A and Author B) evaluated all titles and abstracts. Full-text articles were assessed for eligibility using predefined inclusion criteria. In cases of disagreement, a third reviewer (Author C) resolved the conflict through discussion and consensus. This review followed the PRISMA 2020 guidelines and was registered in PROSPERO (CRD42024506195). A total of eight studies were included in the final review, as illustrated in the PRISMA flow diagram (Figure 1).

Results

Table 1 summarizes the outcomes from eight studies exploring the application of CAT in adolescent interceptive orthodontics, focusing on its clinical effectiveness, patient satisfaction, and treatment predictability.

**Table 1:** Data collection table. CAMD: clear aligner therapy in the mixed dentition; FA: fixed appliances; RA: removable acrylic; IF: Invisalign First; RME: rapid maxillary expansion.

Sr. No.	Title	Authors	Year	Study design	Sample size	Methods	Participants	Results	Conclusion
1	Clear aligner therapy in the mixed dentition: Indications and practitioner perspectives [6]	Lynch et al.	2022	Survey-based	181	22-item survey to orthodontists assessing CAMD usage, indications, compliance, and hygiene.	Practicing orthodontists in the USA	CAMD used less than FA for mixed dentition cases; better oral hygiene was perceived with CAMD.	CAMD is emerging as a treatment modality; perceived hygiene benefits but fewer indications compared to FA.
2	Predictability of expansion movements performed by clear aligners in mixed dentition in both arches: a retrospective study on digital casts [7]	Loberto et al.	2024	Retrospective	36	Digital dental casts at three observation periods (T0, T1, T2) were compared using 3D analysis tools.	Children in early mixed dentition, mean age of 8.3 years, treated with clear aligners	Statistically significant transversal increments in both arches, and predictability increased with refinements.	Clear aligners can induce significant transversal changes; predictability varies and requires refinements.
3	Upper arch dimensional changes with clear aligners in the early mixed dentition: A prospective study [8]	Lione et al.	2021	Prospective	23	Transverse interdental width measurements at T1 and T2 using digital models.	Children (mean age of 9.4 years) in early mixed dentition	Significant maxillary arch expansion, greatest at deciduous molars.	Invisalign First System® is effective for maxillary arch development in growing patients.
4	Indication of clear aligners in the early treatment of anterior crossbite: a case series [9]	Staderini et al.	2020	Retrospective	2	Anterior crossbite treated using clear aligners with a 3D setup.	Two children aged 8 years with an anterior crossbite	Anterior crossbite corrected within 5 months; improved overjet and overbite.	Clear aligners are effective for early anterior crossbite correction and are well-tolerated by young patients.
5	Comparison of acceptability of orthodontic appliances in children in mixed dentition treated with removable acrylic appliances and Invisalign first: a cross-sectional study [10]	Kalaoglu and Dumanli	2024	Cross-sectional	40	Acceptance survey for RA and IF appliances; analyzed compliance and appliance comfort.	Children aged 6-13 years undergoing orthodontic treatment	Invisalign First showed higher acceptance and comfort compared to removable acrylic appliances.	Invisalign First provides better compliance and comfort than removable acrylic appliances.
6	Patient and parental satisfaction following orthodontic treatment with clear aligners and Elastodontic appliances during mixed dentition [11]	Dianiskova et al.	2023	Cross-sectional case-control	56	Comparison of satisfaction levels with clear aligners and elastodontic appliances via surveys.	Children aged 6-12 years; mixed dentition cases	High satisfaction reported; clear aligners rated better for school and social life improvements.	Clear aligners are effective and well-accepted in pediatric orthodontics.
7	Efficacy of clear aligners vs rapid palatal expanders on palatal volume and surface area in mixed dentition patients: A randomized controlled trial [12]	Bruni et al.	2024	Randomized controlled trial	41	Comparison of palatal expansion outcomes using digital models before and after treatment.	Children aged 7-9 years with posterior transverse discrepancies	RME showed superior palatal volume increase compared to Invisalign First; significant intermolar width variation observed.	RME outperforms clear aligners for palatal expansion in early orthodontic treatment.
8	Impact of Invisalign® first system on molar width and incisor torque in malocclusion during the mixed dentition period [13]	Lin et al.	2024	Observational	21	Measurement of dental arch changes and incisor torque using digital scans pre- and post-treatment.	Children aged 6-10 years during the mixed dentition period	Significant arch expansion achieved with moderate torque expression rates.	Invisalign First effectively manages arch expansion and incisor alignment.

Studies employed diverse methods, including retrospective analyses, prospective trials, surveys, and cross-sectional designs, ensuring comprehensive exploration of CAT. Sample sizes ranged from two in case series to 181 in orthodontist surveys. Primarily, children aged six to 12 years in mixed dentition stages with orthodontic conditions like maxillary deficiencies, crossbite, and malocclusions were the participants.

The findings showed that CAT effectively induced significant transverse arch expansion and corrected anterior crossbites within short durations (e.g., five months) and high satisfaction rates among patients and parents, often exceeding those for fixed appliances.

A risk of bias assessment was conducted for all included studies based on their study design. Randomized controlled trials were evaluated using the Cochrane Risk of Bias 2.0 tool, observational studies using the Newcastle-Ottawa Scale (NOS), and case series using the National Institute of Health (NIH) Quality Assessment Tool. Overall, most studies demonstrated a moderate to high risk of bias, primarily due to lack of control groups, small sample sizes, and unclear blinding or allocation concealment. These factors were taken into consideration during data synthesis and interpretation.

Meta-analysis

**Figure 2:**
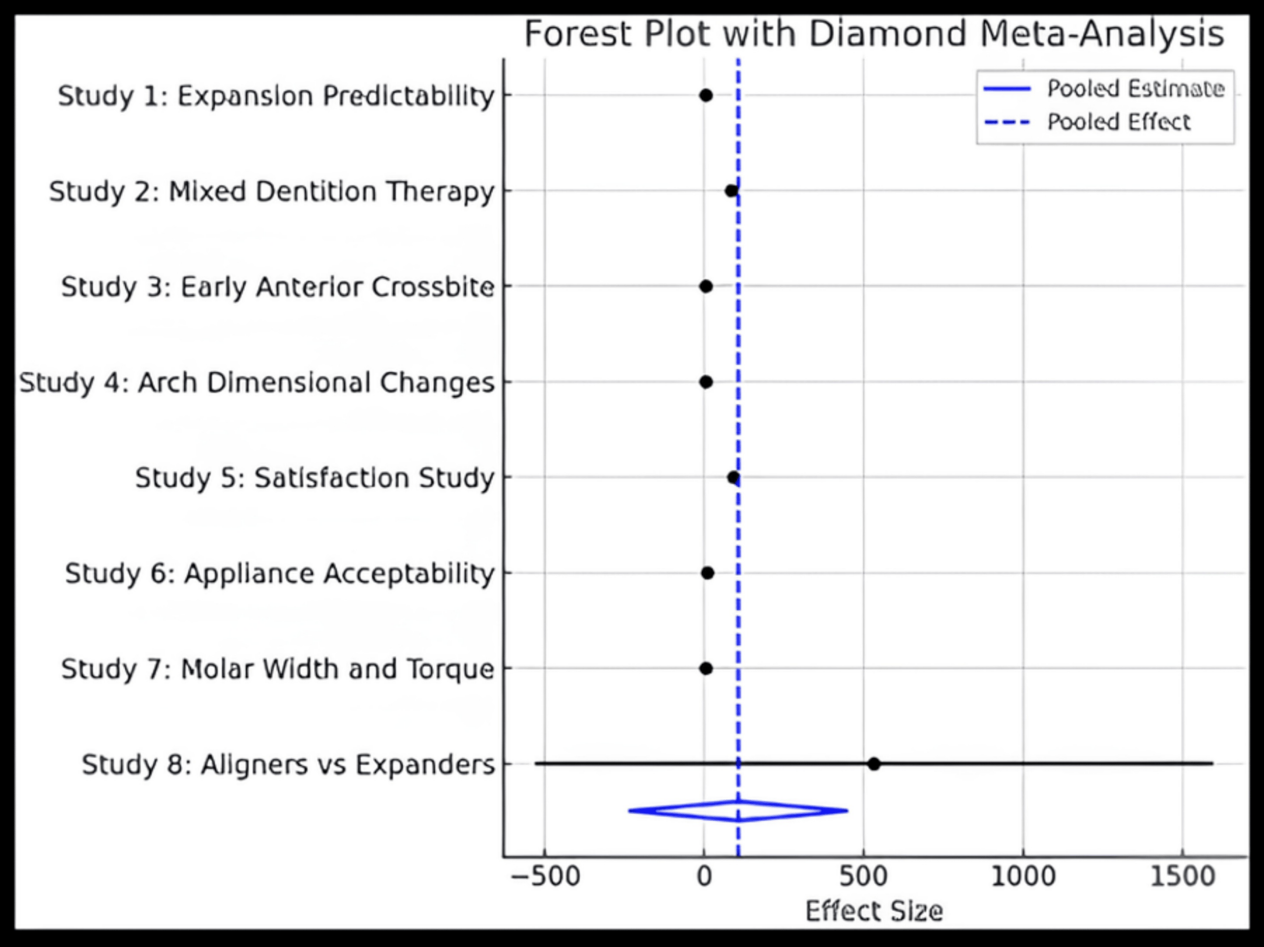
Forest plot with diamond meta-analysis. References [6-13].

The meta-analysis consolidated data from eight studies, each exploring various orthodontic interventions involving CAT in the interceptive phase, such as arch expansion using Invisalign First, anterior crossbite correction, management of class II malocclusions with mandibular advancement features, and comparative analyses of CAT with traditional appliances like rapid palatal expanders, removable acrylic plates, and elastodontic appliances (Table 2 and Figure 2). These studies assessed both clinical outcomes (e.g., transverse width changes, torque control, and palatal volume gain) and patient-reported outcomes, including satisfaction, acceptance, and quality of life. It pointed out that the arch expansion ranged between 3.0 and 3.5 mm on average, with small standard deviations (~0.8-0.9), indicating precise and predictable results in most cases. The satisfaction levels were assessed, and they reported satisfaction scores ranging from 85% to 92%, reflecting the high acceptability of aligners compared to traditional appliances. Palatal volume change assessments demonstrated a significant expansion in some studies; however, the presence of large standard deviations indicated considerable variability in treatment outcomes. These inconsistencies suggest that while clear aligners can effectively induce palatal expansion in many cases, the magnitude and predictability of change may vary across patients. High variability can reduce the statistical power of pooled results and limit the generalizability of findings to broader populations. This may be attributed to differences in patient-specific factors such as age, skeletal growth potential, and individual biological response to aligner forces, as well as treatment adherence, which is particularly variable in younger populations. Consequently, clinicians should interpret average expansion values with caution and consider patient selection and compliance as critical determinants of success with aligner-based expansion protocols. The forest plot, however, collectively indicates a small effect size favoring the therapy.

**Table 2:** Data collection sheet for meta-analysis.

Study	Article 1: Aligners vs. expanders [6]	Article 2: Molar width and torque [7]	Article 3: Appliance acceptability [8]	Article 4: Satisfaction study [9]	Article 5: Arch dimensional changes [10]	Article 6: Early anterior crossbite [11]	Article 7: Mixed dentition therapy [12]	Article 8: Expansion predictability [13]
Sample size	41	21	40	56	23	2	181	36
Outcome considered	Palatal volume change (mm³)	Arch expansion (mm)	Acceptance score (scale 1-10)	Satisfaction (% reporting positive experience)	Arch expansion (mm)	Crossbite correction (months)	Satisfaction (% reporting positive experience	Arch expansion (mm)
Effect size	532.0	3.0	8.2	92.0	3.5	5.0	85.0	3.2
Standard deviation	540.0	0.7	1.5	4.0	0.9	1.0	5.0	0.8

Discussion

CAT has moved well beyond its original application in adult orthodontics and is being used more and more in early interceptive orthodontics, particularly with systems like Invisalign First [3] and Invisalign Mandibular Advancement. The results of this systematic review and meta-analysis present mounting evidence that CAT is effective in treating early mixed dentition malocclusions, especially in obtaining transverse maxillary expansion, correcting anterior and posterior crossbites, and enhancing patient-centered outcomes.

Early treatment with clear aligners is effective in addressing mild to moderate malocclusions, including both posterior and anterior crossbites in patients with mixed dentition. Invisalign First [3] was highlighted for its ability to facilitate arch expansion and correct interceptive orthodontic issues with fewer side effects. These include transient speech alterations, mild discomfort during initial wear, and occasional soft tissue irritation. Increased salivation or dryness may also be reported. In some cases, attachment loss or incomplete tracking can occur, particularly when managing erupting dentition. With proper patient education, monitoring, and refinement, these side effects are generally manageable and do not significantly impact overall treatment success [3]. The orthodontic expansion has emerged as a viable interceptive application of CAT. For instance, Lione et al. (2021) [8] conducted a prospective study involving 23 children (mean age of 9.4 years) in the early mixed dentition stage, treated with the Invisalign First system. Using 3D digital dental models, the authors measured interdental widths at multiple levels (deciduous molars, canines, and permanent molars) before and after treatment. The results demonstrated statistically significant transverse expansion (p < 0.05), particularly at the level of the deciduous molars, supporting the effectiveness of clear aligners in promoting maxillary arch development in growing patients [8].

In class II malocclusion, aligners like Invisalign MA (mandibular advancement) are effective in treating class II malocclusions, with studies reporting improvements in skeletal relationships and airway volumes [5]. Aligners also reduced the need for invasive treatments compared to traditional appliances [14]. Regarding periodontal health, clear aligners have been associated with improved gingival outcomes compared to fixed appliances, as patients consistently report lower plaque accumulation and reduced bleeding indices. For example, studies such as Abbate et al. [15] have shown that aligner users exhibited significantly lower gingival index and plaque index scores than those treated with rapid maxillary expanders (RMEs). Additionally, aligners are generally preferred by patients due to enhanced comfort, easier hygiene maintenance, and fewer soft tissue complications, which may contribute to lower dropout rates and higher compliance during interceptive treatment [16]. Studies also noted lower plaque and bleeding indices among aligner users. However, larger randomized trials are needed to validate aligners' effectiveness in severe cases [17].

Thus, the findings of this systematic review and meta-analysis suggest that CAT shows considerable promise in the interceptive treatment of adolescents, particularly in achieving transverse arch expansion [7] and correcting anterior crossbites [8,9]. Importantly, high levels of patient and parent satisfaction with aligners were consistently reported [11], reinforcing their potential to improve compliance and reduce psychological treatment burdens during formative years. These findings contribute to current knowledge by highlighting the growing viability of CAT in early mixed dentition, an area previously dominated by fixed or removable appliances [14]. The ability of clear aligners to deliver predictable expansion outcomes with fewer side effects, such as soft tissue irritation or molar tipping [14-16], represents a paradigm shift toward more aesthetic, comfortable, and behaviorally compliant treatment options [11].

Despite these promising results, significant limitations both to the reviewed studies and this review itself exist. One of these is the general moderate to high risk of bias overall across studies. Two studies were classified as high risk because of small sample sizes, lack of control groups, and no randomization, characteristics that undermine internal validity. Most studies used subjective self-report measures of satisfaction and compliance [11], with no standardized or validated measures. In addition, blinding of participants and outcome assessors was in many cases lacking, which raises the possibility of observer bias.

Thus, a critical review of the risk of bias across included studies reveals several limitations that must temper interpretation. Some studies had a high risk of bias, primarily due to small sample sizes and lack of control groups [9]. Several others were a moderate risk, lacking randomization or blinding, or relying on self-reported data, especially in studies assessing satisfaction and acceptance. These methodological shortcomings may inflate perceived benefits or obscure true effect sizes. Moreover, heterogeneity in study design, appliances used, and outcome measures limits the generalizability of pooled results.

Additionally, important clinical issues such as long-term stability of arch expansion, skeletal vs. dental effects, and the biomechanical limitations of aligners in growing patients remain underexplored. Few studies addressed class II correction reliability or treatment duration variability [12], which are central to comprehensive interceptive planning. These gaps underscore the need for more robust, multicenter RCTs with standardized outcome reporting and long-term follow-up.

Hence, while CAT offers several patient-centric advantages [11] and emerging evidence of clinical efficacy, its use in adolescent interceptive orthodontics must be guided by cautious optimism, critical appraisal of current evidence, and commitment to further high-quality research.

## Conclusions

This systematic review evaluates the effectiveness of clear aligners in adolescent orthodontics, addressing clinical outcomes, patient satisfaction, and study methodology. Clear aligners demonstrate predictable and significant results, particularly for arch expansion and anterior crossbite correction. Aligners corrected malocclusions within short periods and achieved outcomes comparable to traditional methods. Patients and parents highly appreciated the comfort, aesthetics, and convenience of aligners, with satisfaction rates ranging between 85% and 92%. Limitations of this review include methodological variability and small sample sizes in some studies, such as a case series with two participants, which reduced the reliability of findings. The risk of bias was high in several studies due to a lack of randomization and sample size justification. Larger, well-designed trials with standardized protocols are essential to validate these findings and minimize heterogeneity.
